# Clinical Analysis of TMJ Replacement Using a Customized Prosthesis

**DOI:** 10.3390/jcm14155314

**Published:** 2025-07-28

**Authors:** Sergio Olate, Víctor Ravelo, Gonzalo Muñoz, Carlos Gaete, Rodrigo Goya, Rômulo Valente

**Affiliations:** 1 Center for Research in Morphology and Surgery (CEMyQ), Universidad de La Frontera, Temuco 4780000, Chile; victor.ravelo.s@gmail.com (V.R.);; 2 Department of Oral Diagnosis, Division of Oral and Maxillofacial Surgery, State University of Campinas, Piracicaba 13414-903, SP, Brazil; 3 Grupo de Investigación de Pregrado en Odontología (GIPO), Facultad Ciencias de la Salud, Universidad Autónoma de Chile, Temuco 4780000, Chile; 4 Department of Pediatric Dentistry and Orthodontics, Universidad de La Frontera, Temuco 4780000, Chile; 5 Department of Maxillofacial Surgery, Hospital del Trabajador (ACHS), Santiago 7550196, Chile; 6 Department of Oral and Maxillofacial Surgery, Clinica Universidad de Los Andes, Santiago 7620157, Chile; 7 Department of Oral and Maxillofacial Surgery, Dr. Sotero del Rio Hospital, Santiago 8150215, Chile; 8 Department of Oral and Maxillofacial Surgery, Hospital Getulio Vargas, Recife 50630-060, PE, Brazil; romvalente@yahoo.com.br

**Keywords:** TMJ prosthesis, orthognathic surgery, TMJ disease, patient-specific implant

## Abstract

**Background/Objectives:** This study aims to uncover the variables related to the success of the intervention. **Methods:** A retrospective study was conducted on patients who underwent joint replacement surgery utilizing a customized alloplastic system between 2018 and 2023, comprising subjects with complete records for both the planning and follow-up phases. The Student’s *t*-test was applied with a significance threshold of *p* < 0.05. **Results:** Forty-eight subjects were admitted for initial analysis, and 31 subjects were evaluated with a minimum follow-up of 1 year and a maximum of 7 years, with a mean age of 36.37 ± 15.53. The TMJ diagnosis was mainly with degenerative TMJ disease, followed by ankylosis and craniofacial syndromes, and an average of 2.1 ± 1.2 previous surgeries were noted. Degenerative joint disease correlated with increased pain (*p* < 0.0001) and a higher prevalence of prior joint surgery (*p* < 0.0001). Thirty-one subjects were followed up with 47 prostheses installed; 74.4% underwent complementary surgery with other facial osteotomies. Significant improvements (*p* < 0.0001) were observed when comparing pain levels pre- and postoperatively, with a decrease from 5.5 (±2.3) to 2.2 (±0.4). Concerning the interincisal opening, there was a significant increase (*p* < 0001) from 25.85 (±10.2) mm to 35.93 (±4.2) mm in mouth opening. TMJ replacement treatment is efficient and effective, demonstrating stability in follow-up assessments for up to 7 years. **Conclusions:** The indications for replacement are diverse and may benefit patients who have not yet progressed to end-stage TMJ disease.

## 1. Introduction

The use of temporomandibular joint (TMJ) replacement prostheses has been steadily increasing. Some analyses have shown that the volume of interventions for TMJ replacement by joint prostheses will increase by more than 60% in the coming years [1]. The market has provided answers to this demand with the entry of new players presenting a wide range of joint prostheses [2] built in different countries based on the concept of a component for the fossa and a component for the condyle.

The indication for the use of joint prostheses is currently in agreement with international standards defined by patients and practitioners. Controversies have emerged regarding the indications for surgery in children and teenagers [3] and the optimal timing for its implementation. Early intervention may prevent significant changes in mandibular features and the stability in maxillomandibular function, and overall quality of life [4]. The TMJ degenerative disease, for example, shows the progressive reduction in patients’ functional capabilities and quality of life, making the time from the onset of symptoms to the decision to use the prosthesis critical to treatment success [5].

The use of the TMJ prosthesis is typically related to degenerative TMJ disease, management of facial malformations, and conditions such as trauma and ankylosis. At least half of the cases require complementary orthognathic surgery [6], which involves a highly specialized network for surgical planning and development. It is always important to note that patients’ functionality prior to joint replacement surgery is reduced, showing restrictions in several functional activities such a reduction in mandibular movement, changes in the maxillomandibular anatomy of the lower third of the face, and the presence of pain [7,8], thereby presenting a complex challenge for the comprehensive restoration of the patient’s function and quality of life.

The aim of this retrospective study was to perform a mid- and long-term clinical analysis of the function observed in patients who underwent unilateral or bilateral joint replacement, as well as the characteristics of its planning and surgical execution. Our hypothesis is that patients who undergo unilateral or bilateral temporomandibular joint (TMJ) replacement can show significant improvements in functional outcomes, such as pain reduction and increased mouth opening, in the medium and long term.

## 2. Materials and Methods

A retrospective study evaluated the clinical condition of 48 patients treated unilaterally or bilaterally with TMJ prostheses. The study was conducted in accordance with the Declaration of Helsinki and approved by the Institutional Review Board under protocol 075/23 Universidad de La Frontera (10 August 2023).

We used an Excel spreadsheet to evaluate the pre- and post-surgical clinical data. A convenience sample of patients aged over 18 years who were candidates for joint replacement with a prosthesis was included. Individuals with incomplete medical records or follow-up of less than 12 months will be excluded. Patients showing diagnosis, preoperative data, and planning for TMJ replacement were included as the primary group; subsequently, only patients with at least one year of follow-up (S.O., V.R., G.M.) were included as the secondary group.

The patients who underwent surgery from 2018 to 2023, with a minimum of one-year follow-up, were included in the study. All surgeries adhered to a standardized protocol utilizing the same prosthetic system (Enterprises, Artfix Implants, Pinhais, PR, Brazil), which comprises a fossa component made of ultra-high molecular weight polyethylene (UHMWPE) bonded to a titanium alloy (Ti-6Al-4V ELI) alongside a condylar component featuring a ramus segment fabricated from titanium alloy (Ti-6Al-4V ELI) and a condylar head composed of a cobalt-chromium metal alloy (Co-28Cr-6Mo). All prosthetic systems were customized using a software system, from record acquisition to planning, printing, and device installation.

The analysis included preoperative and postoperative variables such as diagnosis, pain status, dietary habits, maxillomandibular function, and the use of other osteotomies. For the follow-up stage, variables such as pain, kind of diet, opening and closing conditions, among others, were studied.

The data analysis was performed with Graph Prism software version 10.4.1. The Shapiro–Wilk test was used for the normal distribution analysis. The Student’s *t*-test was used to evaluate and compare the variables, considering the value of *p* < 0.05 as a significant difference.

The G*Power software (version 3.1.9.6) was used to perform the statistical power and effect size analysis for the pre- and postoperative samples.

## 3. Results

Of the 48 joint prosthesis candidates (Table 1), degenerative joint disease (52.08%) was the most frequent, followed by ankylosis (14.58%), craniofacial syndrome (12.5%), condylar tumor (12.5%), and idiopathic condylar resorption (8.33%). In relation to the affected joint side, subjects with ankylosis, craniofacial syndrome, and condylar tumor had a greater incidence of unilateral disease (*p* < 0.06), whereas subjects with degenerative joint disease had a higher prevalence of involvement in both TMJs (*p* < 0.03).

Forty-eight subjects treated with TMJ replacement, with a mean age of 36.37 ± 15.53, were included. When analyzing the diagnosis of facial deformity (Table 2), more subjects had facial asymmetry, followed by subjects with class II deformity (CII) and subjects with class III deformity (CIII). The TMJ diagnosis showed that 93.75% of the CII subjects presented degenerative TMJ disease, while in the subjects with facial asymmetry, 34.48% presented degenerative TMJ disease, followed by 24.13% with ankylosis and 20.68% with craniofacial syndromes. CIII facial deformity was observed only in subjects with condylar tumors.

Concerning pain and prior surgeries, 70% of the subjects exhibited pain with an opening range of 27.76 ± 12.68 mm and an average of 2.1 ± 1.2 previous surgeries, with arthrocentesis, discopexy, and discectomy being the most frequent. Degenerative joint disease was associated with a greater presence of pain (*p* < 0.0001), an average VAS scale of 5.8 ± 2.4, and a higher prevalence of previous joint surgeries (*p* < 0.0001).

Forty-eight subjects were initially included at the diagnostic stage. However, 35.4% were excluded due to a lack of 1-year postoperative follow-up data. Therefore, an analysis of surgical details and follow-up outcomes for at least 1 year was performed in the remaining 31 subjects.

The average age of the 31 subjects who underwent joint replacement surgery was 37.16 ± 17.82 years; 5 (16.12%) were male, and 26 (83.87%) were female. Regarding facial diagnosis, 19 had facial asymmetry, and 12 had a CII facial deformity. In the TMJ diagnosis group, 18 had osteoarthritis, 5 had unilateral condylar tumors, 3 had ankylosis, 3 had hemifacial microsomia, 1 had rheumatoid arthritis, and 1 had idiopathic condylar resorption; 67.74% had significant restrictions in oral opening.

Sixteen bilateral joint replacements and 15 unilateral replacements were performed, for a total of 47 prostheses. Of the 31 subjects, 77.41% had complementary surgery with other facial osteotomies. Of the 16 subjects who required bilateral prostheses, 11 underwent Le Fort I maxillary surgery, and 6 underwent genioplasty. Of the 15 unilateral joint replacements, 10 required maxillomandibular surgery, and 3 required only mandibular surgery. It was noted that subjects with bilateral joint replacements have a significantly higher rate of Le Fort I osteotomy (*p* < 0.003).

Total surgery time was 9.05 ± 3.2 h for bilateral prostheses and 7.8 ± 2.2 h for unilateral prostheses. The average follow-up was 38.09 ± 26.6 months with a range of 12 to 104 months. After 12 months, 38.7% of subjects presented some type of localized facial nerve alteration, with painless paresthesia being the most common. In addition, one subject experienced muscle pain, and another presented a postoperative occlusal change, which resolved at the follow-up visit with dental treatment. The remaining subjects did not experience pain-related problems or neurosensory disturbances. All subjects underwent postoperative physiotherapy, and no further surgery was required to correct or modify any aspect of the TMJ prosthesis. Regarding the assessment of pain using the VAS scale (Table 3), we observed significant differences (*p* < 0.0001) when comparing pain in the pre- and postoperative pain levels, with a reduction from 5.5 ± 2.3 to 2.2 ± 0.4. In relation to the interincisal opening, there was a statistically significant increase (*p* < 0001), improving from 25.85 ± 10.2 mm to 35.93 ± 4.2 mm after follow-up. No prosthesis was removed, and no infectious process was observed on the follow-up.

A post hoc power analysis was conducted to compare preoperative and postoperative outcomes (maximum incisal opening and pain levels) in patients treated with temporomandibular joint prostheses. Using a paired-samples t-test with a total sample size of 31 subjects, the achieved statistical power was 0.86. This indicates that the study had an 86% probability of detecting a true moderate effect, suggesting that the sample size was adequate to support the reliability of the observed clinical differences. Additionally, a sensitivity analysis was performed using a significance level of 5% and a power of 86%, which yielded a minimum detectable effect size (dz) of 0.5, corresponding to a moderate effect according to previous studies, which mention a medium effect size. The effect size should be 0.3, not 0.5 [9]. These results indicate that the study had sufficient statistical power and effect size sensitivity to detect pre- to postoperative changes in maximum incisal opening and pain levels in patients treated with temporomandibular joint prostheses.

## 4. Discussion

The results of this study showed a stable surgical protocol with positive outcomes, with an average follow-up of 38.09 ± 26.6 months and a range of 12 to 104 months, extending in one patient for 7 years.

Today, TMJ prostheses are a reliable and efficient option for managing joint-destructive diseases. All management of TMJ dysfunction requires initial non-surgical or conservative treatment [10,11] because a large percentage of patients will be able to improve their functional condition with the initial therapy. However, a smaller group of patients will have repeated failures with these techniques and will require early and advanced surgical management to obtain favorable results and mitigate potential complications [12,13]. The subjects included in this study underwent surgery beginning in 2018, indicating significant time for managing.

Patients treated with TMJ prostheses have a history of joint disease; Amarista et al. [14] reported on a series of 28 patients undergoing replacement surgery for TMJ ankylosis or fibrous ankylosis, where 75% of the patients had one or more TMJ surgeries before prosthesis installation, the main etiology being trauma or degenerative TMJ disease. In this series, there was an average of 2.1 ± 1.2 previous joint surgeries, and no statistical relationship with patient behavior was observed. In another sequence of 129 patients undergoing joint surgery with a discectomy, 75% were successful during follow-up. In contrast, 11.7% of patients were indicated for joint replacement on average 7.8 years after the discectomy [15]. In these cases, the quality of life of patients who required TMJ prosthesis was not documented; however, it is possible to infer that it was suboptimal, leading to the need for prosthetic intervention.

Other studies have shown that patients with one or more previous surgeries are more likely to have pain even after joint replacement [15,16]. In our sample, only one subject had increased pain after prosthesis installation, likely associated with previous conditions related to long-time conservative treatment, bad selection of treatments in the surgical phase, or psychiatric and pain factors. In addition, Gerber and Saeed [17] noted that in 73 patients treated with joint replacement, severe pain decreased from 50.7% to 6.8%, and moderate pain decreased from 27.4% to 9.6%, demonstrating that some cases maintain pain even after TMJ prosthesis. In our series, the intensive use of physical therapy and occlusal controls reduced the pain condition, and although there was a reduction from 5.5 (±2.3) to 2.2 (±0.4), the mild postoperative pain could be controlled by the patient until the time of this study.

Clinical studies have revealed moderate to advanced symptomatology in cases of degenerative TMJ disease, which do not appear to correlate precisely with a terminal condition of the TMJ [18]. For example, in a cohort of 39 patients with inflammatory disease and connective tissue disorders, a preoperative mouth opening greater than 20 mm and moderate pain were noted; the installation of TMJ prostheses in these patients proved to be highly successful, yielding functional improvements alongside facial morphology correction [19]. In our series, pain presented a significant reduction, and mandibular mobility also improved significantly (*p* < 0001) with an opening increase from 25.85 (±10.2) mm to 35.93 (±4.2) mm. In those cases, the preoperative functional limitations cannot be deemed totally severe; nevertheless, the use of a prosthesis enhanced the patient’s overall condition. It is possible to think that in cases with advanced damage of the TMJ, the TMJ replacement may be necessary; the advances cases usually show more pain in the long term, and psychological conditions could be involved in a reduced response of the patient to the prosthesis performance.

This study includes patients treated exclusively with customized prostheses using a single system (Enterprises, Artfix Implants, Pinhais, PR, Brazil) and a standardize protocol and methodology for planning and surgery. Kanatsios et al. [20] conducted a study to compare the clinical condition between stock and customized systems, concluding that both can provide patients with adequate function. They included patients between 45 and 55 years old who were mainly unilateral, and their most frequent complications were associated with nerve injury. We consider that using customized prostheses improves surgical management, using an ideal implant for TMJ replacement, in agreement with other authors [21]. Complementary orthognathic surgery is a common procedure for using TMJ prostheses [6]. In this series, 77.41% of the patients underwent other maxillomandibular osteotomies, demonstrating that the complexity of the cases is optimized with virtual planning and patient-specific implants with good postoperative outcomes [22].

TMJ treatment with custom-made prosthetics is tailored to each individual patient. The manufacturing process plays a crucial role in the success of the procedure, helping to minimize both biological demands and economic costs. It has been shown that different commercial brands of prosthetic systems may exhibit differences in microstructure and electromechanical properties, potentially affecting the success and corrosion resistance of current systems [23]. In this study, a unique system was used to define the planning protocol as one of the study variables, and the system used has been presented in another publication [6]. Of the 47 prostheses installed and with an average follow-up of 38.09 ± 26.6 months (12 to 104 months), no need for removal, exchange, or admission to revision surgery was observed. However, some publications show conditions that could be related to this situation [24,25].

The publication by Olate et al. [6] in 2025 reports the indication for TMJ prosthesis due to the need for removal of a previously installed prosthesis; this may be associated with metal allergy, infection, presence of heterotopic bone, or failure in the manipulation and installation of the prosthesis [26]. Our series of patients was planned and treated by four surgeons with more than 15 years of experience in using and managing TMJ prostheses, which may contribute to treatment success [4]. In addition, in the case of high volume of interventions for TMJ prostheses, there is a significantly better postoperative performance [27]. This research provides relevant information for clinical diagnoses related to treatments involving joint prostheses, as well as complementary surgical procedures that can be incorporated into surgical planning with the aim of restoring facial aesthetics and function. Additionally, the outcomes observed after treatment were favorable in the short and medium term, as all subjects showed increased oral opening and reduced pain levels.

Among the limitations of this study, we observed a reduction in our initial sample size, which is associated with a decrease in statistical power. Therefore, it is necessary to increase the sample size and extend the follow-up period to better assess prosthesis survival and associated complications.

## 5. Conclusions

We can conclude that TMJ replacement using a customized alloplastic implant, as described in this study, has proven to be an efficient and effective treatment option, demonstrating favorable clinical outcomes. The indications for joint replacement are diverse and may be justified in patients who have not yet reached the final stage of temporomandibular joint (TMJ) disease, especially when dramatic changes in mandibular morphology and lack of occlusal balance are observed, as in autoimmune disease. In our study, we observed that joint replacement is a safe and stable alternative that reduces pain and increases mouth opening over an average follow-up period of 38.09 ± 26.6 months.

## Figures and Tables

**Table 1:** Descriptive analysis of the clinical characteristics at the diagnostic stage and their relationship with joint pathologies.

	Degenerative Diseases (n:25)	Condylar Resorption (n:4)	Craniofacial Syndrome (n:6)	Ankylosis (n:7)	Tumors (n:6)		
	N	N	N	N	N	*p* < 0.05	Effect Size
**Unilateral TMJ**	9	2	5	4	6	0.06	0.14
**Bilateral TMJ**	16	2	1	3	0	0.03 *	0.19
**Presence of joint pain**	25	2	0	2	2	0.0001 *	0.72
**Previous TMJ surgeries**	23	4	3	6	2	0.0001 *	0.64

*: Statistical significance.

**Table 2:** Description of the diagnosis of pathologies affecting the TMJ in relation to sex and facial deformity.

	Sex		Facial Deformity		
	M (n:11)	F (n:37)	CII (n:16)	CIII (n:3)	Asymmetry (n:32)
**Degenerative disease**	2	23	15	0	10
**Idiopathic condylar resorption**	2	2	1	0	3
**Craniofacial syndrome**	2	4	0	0	6
**Ankylosis**	4	3	0	0	7
**Condylar tumor**	1	5	0	0	6

M: male, F: female, CII: skeletal class II, CIII: skeletal class III.

**Table 3:** Comparative analysis of pain and interincisal opening at the diagnostic (T0) and postoperative (T1) stages.

	T0	T1		
	X	X	*p* < 0.005	Effect Size
**Pain VAS**	5.5 ± 2.3	2.2 ± 0.4	0.0001 *	0.70
**Incisal opening**	25.85 ± 10.2	35.93 ± 4.2	0.001 *	0.92

VAS: visual analogy scale; *: Statistical significance.

## Data Availability

The data are available upon request from the corresponding author.
